# Supporting patient access to medicines in community palliative care: on-line survey of health professionals’ practice, perceived effectiveness and influencing factors

**DOI:** 10.1186/s12904-020-00649-3

**Published:** 2020-09-24

**Authors:** Sue Latter, Natasha Campling, Jacqueline Birtwistle, Alison Richardson, Michael I. Bennett, Sean Ewings, David Meads, Miriam Santer

**Affiliations:** 1 School of Health Sciences, University of Southampton, Building 67, Highfield, Southampton, SO17 1BJ England; 2 Leeds Institute of Health Sciences, University of Leeds, Worsley Building, Clarendon Way, Leeds, LS2 9NL England; 3 University Hospitals Southampton NHS Foundation Trust, Southampton General Hospital, Tremona Road, Southampton, SO16 6YD England; 4 Faculty of Medicine, University of Southampton, Building 85, Southampton, SO17 1BJ England; 5 School of Medicine, University of Leeds, Worsley Building, Leeds, LS2 9JT England; 6 Faculty of Medicine, University of Southampton, Aldermoor Health Centre, Aldermoor Close, Southampton, SO16 5ST England

**Keywords:** Palliative supportive care, Community, Medicines, Access to health care, Non-medical prescribing

## Abstract

**Background:**

Patient access to medicines at home during the last year of life is critical for symptom control, but is thought to be problematic. Little is known about healthcare professionals’ practices in supporting timely medicines access and what influences their effectiveness. The purpose of the study was to evaluate health professionals’ medicines access practices, perceived effectiveness and influencing factors.

**Methods:**

On-line questionnaire survey of health care professionals (General Practitioners, Community Pharmacists, community-based Clinical Nurse Specialists and Community Nurses) delivering end-of-life care in primary and community care settings in England. Quantitative data were analysed using descriptive statistics.

**Results:**

One thousand three hundred twenty-seven responses were received. All health professional groups are engaged in supporting access to prescriptions, using a number of different methods. GPs remain a predominant route for patients to access new prescriptions in working hours. However, nurses and, increasingly, primary care-based pharmacists are also actively contributing. However, only 42% (160) of Clinical Nurse Specialists and 27% (27) of Community Nurses were trained as prescribers. The majority (58% 142) of prescribing nurses and pharmacists did not have access to an electronic prescribing system. Satisfaction with access to shared patient records to facilitate medicines access was low: 39% (507) were either Not At All or only Slightly satisfied. Out-of-hours specialist cover was reported by less than half (49%; 656) and many General Practitioners and pharmacists lacked confidence advising about out-of-hours services. Respondents perceived there would be a significant improvement in pain control if access to medicines was greater. Those with shared records access reported significantly lower pain estimates for their caseload patients.

**Conclusions:**

Action is required to support a greater number of nurses and pharmacists to prescribe end-of-life medicines. Solutions are also required to enable shared access to patient records across health professional groups. Coverage and awareness of out-of-hours services to access medicines needs to be improved.

## Background

Timely patient access to medicines during the last year of life is essential for control of symptoms. Patients are commonly managing symptoms at home and may experience extensive barriers to accessing essential medicines: our recent study suggested prescription, dispensing, supply and associated information given about medicines^1^ are difficult, complex, demanding, lack co-ordination, and involve a multiplicity of professionals [1]. Problems were identified with each element of the access process – ranging from the time-consuming nature of obtaining new prescriptions, lack of pharmacy stock of medicines required, to limited medicines information and misconceptions [1]. There is very limited research internationally on experiences of accessing medicines for community palliative care, but a few small studies are suggestive of problems. Difficulties accessing medicines and medicines-related information, especially out-of-hours, were cited by both palliative care service users and health care professionals in a recent study of medication management for community palliative care patients in Australia [2]. In a small study [3] of 22 palliative care patients’ access to medicines in the community in Ireland, multiple points of system failure were found, including: patients unable to attend the General Practitioner (GP) (family doctor) for a prescription, community nurse inability to contact GP for a prescription, and lack of appropriate pharmacy stocks. Difficulties and delays in accessing medicines is likely to adversely affect symptom control, quality of life and use of unplanned and out-of-hours services.

In England, community palliative care health services are free at the point of delivery and may be offered to patients living at home by either generalist and / or specialist health professionals. Generalists include the patient’s family doctor (General Practitioner), community nurses who provide services for the General Practitioner practice, community pharmacists (high street chemists) and primary care-based pharmacists, working at the General Practice site. Specialists include community-based nurse specialists, who are either employed by a local hospice or a local community or acute care Trust. Recent national guidance in England on service delivery for end-of-life care (in UK policy and practice this commonly equates to the last year of life [4]) recommends the provision of multi-practitioner care, delivered by those who have the skills and expertise to meet patient needs, which includes access to medicines [4]. At the same time, there are indications that sectors of the workforce critical to palliative care prescribing, dispensing, supply and early information-giving could be under-utilised. UK legislation, in common with an increasing number of other countries worldwide [5], permits nurses and pharmacists who have undertaken the requisite prescribing training course to prescribe medicines directly to patients. But data suggest prescribing by specialist palliative care nurses has not been exploited to its full potential [6]. Little is known about why nurses’ roles in prescribing in this area remain under-developed and wider evidence about the impact on access to medicines is lacking. In addition, community pharmacists’ - pharmacists based in retail pharmacies or ‘chemist shops’ - expertise in palliative care medicines optimisation has been found to be under-utlilised [7, 8]. In England, community pharmacy palliative care medicines services have been commissioned for a number of years; typically these involve some pharmacies in a local area opting to be paid by the National Health Service to stock a core list of palliative care medicines and / or offer extended opening hours for patients to access these medicines. However, the impact of this initiative on access experiences remains un-evaluated.

A small number of studies in the US [9–11] point to the effectiveness of anticipatory prescribing kits in home hospice care as a way of increasing timely patient access to medicines in the home, but internationally, there is a lack of research into how health professionals and different systems of care delivery shape patients’ experience of access to medicines.

## Methods

### Aim

As part of a larger study [12] our objective was to undertake a large-scale survey to:

1. evaluate community-based health professionals’ interventions to support patient access to medicines
2. identify the factors that influence health professionals’ ability to support patient medicines access
3. evaluate the extent to which the community-based nurse and pharmacist workforce are engaged in interventions to improve patient access to medicines at end-of-life
4. elicit views on how effective professionals perceive their service to be and what influences this

### Design

An on-line questionnaire survey was designed using the Checklist for Reporting Results of Internet E-Surveys (CHERRIES) [13]. A systematic narrative review of international literature evaluating experiences, influences and outcomes of medicines access associated with various forms of delivering palliative care services informed questionnaire design [12]. In light of the limited research in this area, we also consulted with clinical and academic experts in the field to identify key issues. Both paper and on-line versions of the questionnaire were piloted with health professionals (total *n* = 19) inclusive of each of the target groups: GPs, community nurses, clinical nurse specialists, primary care and community pharmacists. Minor amendments were made in light of feedback. The final questionnaire, administered via Online Surveys© (onlinesurveys.ac.uk, Jisc), comprised sections including: roles in access provision, in-hours and out-of-hours medicines provision, inter-professional medicines communication, nurse and pharmacist prescribing, and pain control levels of patients - as a proxy to estimate perceived service effectiveness and factors influencing service effectiveness. Items (1–3 per page) comprised closed-ended response format, including Likert scales, and open-ended items (see Supplementary File: ActMed questionnaire). Piloting indicated that the questionnaire would take no longer than approximately 10 min to complete.

### Sample

The target sample comprised representatives from the main professional groups (generalist and specialist) providing community-based end-of-life care in England: medical General Practitioners (GPs), pharmacists working in a community pharmacy (community pharmacists) pharmacists who are employed part-time in GP practices (primary care pharmacists), nurse specialists (Clinical Nurse Specialists) in end-of-life care, and nurses (Community Nurses) delivering a range of care in patients’ homes, including palliative care.

### Recruitment process and access

A survey website link was distributed via e-mail to GPs and pharmacists via research leads employed as part of local networks of research active GP practices and community pharmacists in England (National Institute for Health Research Clinical Research Networks, which fund and manage infrastructure to support the conduct of research). Based on estimated numbers of health professionals in a typical Clinical Research Network and our target response of 200 in each professional group, the link was distributed to GPs in registered research active General Practices in four Clinical Research Networks (two in the north of England, two in the south), and to pharmacists in all 15 Clinical Research Networks in England. Distribution to pharmacists occurred via various local pharmacist networks operating within the Clinical Research Networks. Clinical leads for all hospices in England with teams of Clinical Nurse Specialists delivering community-based palliative care (*n* = 146 /167 hospices in England) distributed the survey via e mail to their Clinical Nurse Specialist team, as did clinical leads in seven community trusts (local community care delivery organisations in England) that we identified employ Clinical Nurse Specialists directly. Based on estimated numbers of Community Nurses in a typical community trust, local collaborators in four community trusts (two in northern, one in southern, and one in eastern England) distributed the e-mail survey link to all Community Nurses in the trust. In addition, the survey link was posted on relevant interest groups’ websites and in newsletters, for example, Association of Supportive and Palliative Care Pharmacy, eHospice and the Association for Prescribers. For GP practices, community pharmacies and community trusts, organisational level participation was captured via National Institute for Health Research processes, which contributed to overall payments to the Clinical Research Networks and to GP practices who participated.

### Patient and public involvement

The study research questions were derived from patient experiences reported in our previous research [1] and through consultation with local groups of palliative care patients and unpaid carers caring for a family member in the last year of life. The study team includes a Public and Patient Involvement co-applicant, and the Study Scientific Committee includes two Patient and Public Involvement members, whose views have helped shape the survey questions. The study Patient and Public Involvement representatives will all be offered a lay summary of the study findings.

### Data collection

Data were collected July – October 2018. Up to three reminders were distributed to maximize responses.

### Analysis

Quantitative data were analysed using descriptive statistics. For the analysis of the pain control data, we conducted tests of differences in proportions comparing Q1 (current pain levels) and Q2 (estimated pain levels with improvement to medicines access) responses. We also created a weighted estimate from Q1 data (pain level estimates across a typical 100 end-of-life patients who use your service) by multiplying the pain category (No = 0; Mild = 1; Moderate = 2; Severe = 3) by the proportion of individuals respondents estimated would be in those categories (thus, higher values represented higher levels of pain). We then conducted linear regression with the weighted variable as the dependent variable controlling for the role of the respondent. Statistical analyses were conducted using Stata®. A directed content analysis approach [14] was undertaken (NC and JB) to analyse free text responses, including quantification.

## Results

### Respondents

A total of 1327 responses from eligible healthcare professionals were received: 499 GPs, 389 Clinical Nurse Specialists, 219 community pharmacists, 151 primary care pharmacists (30 were employed as both community pharmacists and primary care pharmacists) and 99 Community Nurses. Missing data were very low (< 1% for the majority of questions); all responses provided were used (i.e., no case-wise deletion) and no imputation was carried out. All percentages are calculated from responses provided (i.e. excluding missing data).

### Medicines access practices

#### Methods of providing new prescriptions in-hours

Access routes to new prescriptions for palliative care medicines during working hours are shown in Table 1. (These medicines were defined as regular and as necessary medicines, administered via all routes for symptom management during the last year of life (excluding ‘Just in Case’ boxes^2^)).

**Table 1:** Routes provided for access to new prescriptions during working hours

Which of the following are you able to provide for patients to obtain new prescriptions during working hours? Please select all options you use:					
	CNS N (%)	CN N (%)	GP N (%)	PCP N (%)	CP N (%)
Personal home visits	349 (92)	82 (93)	484 (98)	52 (43)	49 (35)
Telephone consultations	335 (88)	78 (91)	498 (100)	134 (96)	147 (85)
Email consultations	79 (24)	8 (10)	169 (35)	32 (28)	63 (43)
GP practice appts.	168 (51)	43 (56)	496 (100)	122 (91)	44 (38)
Community pharmacy appts.	37 (12)	11 (15)	118 (29)	28 (29)	131 (73)
Referral to nurse prescriber	194 (58)	69 (78)	286 (65)	102 (78)	101 (62)
Referral to pharmacist prescriber	64 (20)	21 (26)	149 (34)	66 (65)	81 (53)
Referral to GP	375 (97)	92 (96)	351 (95)	147 (100)	185 (93)

Percentages calculated as those responding yes out of those applicable (i.e., discounting NAs)

NOTE: different numbers of “NA” recorded throughout so percentages have different denominators

Key: *CNS* Clinical Nurse Specialist, *CN* Community Nurse, *GP* General Practitioner, *PCP* Primary Care Pharmacist, *CP* Community Pharmacist

Most nurses and GPs were providing home visits for patients to obtain new prescriptions, high numbers of all professional groups were also providing consultations by telephone, and a significant minority also reported using e-mail. Over 93% of all professional groups used referral to a GP as a route for patients to access medicines; large proportions of all professionals also cited referral to a nurse prescriber. Table 1 also shows a relatively high level of engagement in helping to provide new scripts by primary care pharmacists. 52% (79) of the primary care pharmacist sample also reported advising patients / carers about palliative care medicines (43% (65) did not) and 63% (95) reported engagement in systematically reviewing medicines prescribed for palliative care patients (32% (49) did not).

Overall, many respondents were satisfied with their ability to support patients to obtain new prescriptions during working hours: 57% (762) were Extremely or Very Satisfied, especially GPs (77% 386); however, 43% (567) of the sample were Somewhat, Slightly or Not At All Satisfied.

### Nurse and pharmacist prescribing

Of nurse and pharmacist respondents, 42% (160) of Clinical Nurse Specialists, 27% (27) of Community Nurses, 76% (114) of primary care pharmacists and 16% (36) of community pharmacists were qualified as Independent Prescribers. The most common reasons for not training as a prescriber were the cost of training (31% 155), lack of employer / colleague support (24% 122), no backfill of their post available whilst attending training (18% 92) and no designated clinically-based trainer available (16% 80), which is a requirement for UK nurse and pharmacist prescriber trainees.

Of the nurse and pharmacist respondent groups, Clinical Nurse Specialists prescribed most frequently, with two thirds (66%; 86) prescribing at least 2–3 times per week, whereas two thirds (64%; 14) of the Community Nurses, primary care pharmacists (69%; 60) and community pharmacists (60%; 12) only prescribed once per month or less. Analgesics, anti-emetics and laxatives were identified as the most frequently prescribed medicines by all of the nurse and pharmacist prescriber professional groups. For all respondent groups, the majority (84% 206) prescribed Controlled Drugs (controlled substances), ranging from 96% (125) of Clinical Nurse Specialists to 69% (60) of primary care pharmacists.

The majority (58% 142) of prescribing nurses and pharmacists were not able to use an electronic prescribing system (whereby details of prescribed medicine/s are entered electronically and where scripts can be sent direct to a pharmacy for dispensing to the patient) (Table 2).

**Table 2:** Nurses’ and pharmacists’ ability to prescribe electronically

Are you able to prescribe via an electronic system?					
	Total N (%)	CNS N (%)	CN N (%)	PCP N (%)	CP N (%)
No – pad only	142 (58)	122 (94)	14 (64)	68 (78)	4 (20)
Yes – with transfer to pharmacy	74 (30)	2 (1.5)	4 (18)	17 (20)	11 (55)
Yes – no transfer to pharmacy	28 (11)	6 (4.6)	4 (18)	2 (2.3)	5 (25)

Key: *CNS* Clinical Nurse Specialist, *CN* Community Nurse, *GP* General Practitioner, *PCP* Primary Care Pharmacist, *CP* Community Pharmacist

The vast majority of Clinical Nurse Specialist prescribers in particular, were restricted to writing prescriptions by hand via a paper prescription pad only. Only a minority of all respondent groups were able to prescribe electronically *and* transfer this electronically to the pharmacy.

### Out-of-hours services for medicines access

Asked if there was Clinical Nurse Specialist service provision 7 days a week in their area, overall 49% (656) responded Yes, 18% (239) stated No provision and 33% (424) were not aware whether there was provision or not. Of note, 76% (166) of community pharmacists, 72% (109) of primary care pharmacists and 33% (167) of GPs were not aware. 20% (76) of Clinical Nurse Specialist respondents reported covering 6.30 pm-8.00 am weekdays and 71% (276) reported covering weekends and Bank Holidays; for Community Nurses, cover was 43% (43) and 67% (66) respectively.

Respondents were also asked how effective Clinical Nurse Specialist 7 days a week cover was at facilitating out-of-hours access to medicines for patients. Overall, 43% (280) rated Clinical Nurse Specialist cover as Extremely or Very Effective; 36% (235) reported Somewhat and 22% (141) only Slightly or Not At All effective. Analysis of comments indicated that Clinical Nurse Specialists’ ability to prescribe medicines seemed to be critical in their perceived effectiveness, and, for some, this also interacted with access to pharmacy stocks to influence speed of access.

Asked about confidence in their ability to advise patients how to best access palliative care medicines out-of-hours, nurses – in particular Clinical Nurse Specialists– tended to rate themselves as most confident. 79% (307) of Clinical Nurse Specialists were Extremely or Very confident, as were 61% (61) of Community Nurses. Pharmacists were less confident, with 30% (45) of primary care pharmacists and 39% (86) of community pharmacists rating themselves as Extremely or Very confident. Although 47% (236) of GPs reported being Extremely or Very confident, 35% (177) were only Somewhat confident and 18% (86) were Slightly or Not At All confident.

### Community pharmacy commissioned service provision

Table 3 shows the provision of commissioned palliative care medicines services by community pharmacist respondents (*n* = 219).

**Table 3:** Provision of commissioned community pharmacy palliative care medicines services

If you are a community pharmacist in a community pharmacy, do you provide an enhanced service for palliative care (e.g. on-demand availability of specialist drugs)?		
	Yes	No
Commissioned service^a^	67 (31)	148 (68)
*Of those with a commissioned service*		
Stocking a locally agreed list of core palliative care medicines	62 (93)	5 (7.5)
OOH availability of palliative care medicines from your pharmacy	32 (48)	35 (52)
OOH availability of palliative care medicines from other linked pharmacies in your area	19 (28)	48 (72)
Provision of info on the service to other pharmacy contractors and HCPs to signpost patients to the service	47 (70)	20 (30)
Provision of info on the service to patients and carers directly	41 (61)	26 (39)

^a^Four (4; 1.8%) missing

Key: *OOH* Out-of-hours, *HCPs* Health Care Professionals

The vast majority of services included stocking a core list of agreed palliative care medicines; a smaller proportion – approximately half – reported providing out-of-hours medicines from their own pharmacy and 28% (19) via a linked pharmacy in their area. Around two thirds of the sample reported providing information on the service to health care professionals (70% 47) and directly to patients and carers (61% 41).

Asked whether they were aware of commissioned services for palliative care medicines, overall 39% (517) of all healthcare professionals stated Yes, 9.2% (122) reported No Provision and 52% (690) were Unaware. GPs in particular lacked awareness (68%; 340). However, where respondents were aware, the vast majority (84% 433) considered that the service facilitates speed of access to medicines for patients.

### Factors influencing medicines access service provision

#### Prescribing competence

Overall, just under two thirds (64% 518) of the sample reported feeling Very (46% 373) or Extremely (18% 145) competent in prescribing palliative care medicines, with 27% (217) feeling Somewhat, and 10% (84) reporting Slightly or Not At All competent. Clinical Nurse Specialist respondents reported greatest competence, with 84% (132) feeling Very or Extremely competent; 70% (347) of GPs reported feeling Very or Extremely competent, with almost one third (29% 142) rating themselves as Somewhat competent. Over one third (40% 10) of Community Nurses reported feeling Very or Extremely competent, with 48% (12) reporting they were Somewhat competent. Pharmacists reported less competence: 22% (24) of primary care pharmacists and 14% (7) of community pharmacists reported feeling Extremely or Very competent.

#### Access to patient records

Experience of access to shared patient records for communicating about medicines is shown in Table 4.

**Table 4:** Access to patient records

Do you have access to shared patient records for communication about medicines access between health professionals? Tick all that you are able to access:															
	CNS			CN			GP			PCP			CP		
	Paper N (%)	Elec. N (%)	No N (%)	Paper N (%)	Elec. N (%)	No N (%)	Paper N (%)	Elec. N (%)	No N (%)	Paper N (%)	Elec. N (%)	No N (%)	Paper N (%)	Elec. N (%)	No N (%)
GP records	22 (5.7)	203 (52)	173 (44)	5 (5.1)	77 (78)	18 (18)	–	–	–	14 (9.3)	145 (96)	2 (1.3)	10 (4.6)	62 (28)	149 (68)
GP OOH records	8 (2.1)	117 (30)	264 (68)	1 (1.0)	60 (61)	38 (38)	72 (14)	349 (70)	86 (17)	13 (8.6)	100 (66)	39 (26)	6 (2.7)	21 (9.6)	192 (88)
CN records	77 (20)	218 (56)	105 (27)	–	–	–	39 (7.8)	281 (56)	186 (37)	7 (4.6)	97 (64)	48 (32)	1 (0.5)	15 (6.9)	203 (93)
Hospice/ palliative care specialist records	–	–	–	3 (3.0)	66 (67)	30 (30)	46 (9.2)	154 (31)	304 (61)	20 (13)	58 (38)	77 (51)	3 (1.4)	12 (5.5)	204 (93)
CP records	2 (0.5)	18 (4.6)	369 (95)	1 (1.0)	8 (8.1)	90 (91)	12 (2.4)	29 (5.8)	458 (92)	4 (3.3)^a^	9 (7.4)^a^	108 (89)	–	–	–
Summary Care Record	23 (5.9)	153 (39)	219 (56)	2 (2.0)	60 (61)	37 (37)	26 (5.2)	417 (84)	59 (12)	6 (4.0)	138 (91)	8 (5.3)	13 (5.9)	198 (90)	8 (3.7)

^a^For those classed as only primary care pharmacists (i.e., not primary care and community pharmacist)

Key: *CNS* Clinical Nurse Specialist, *CN* Community Nurse, *GP* General Practitioner, *PCP* Primary Care Pharmacist, *CP* Community Pharmacist, *Elec* Electronic, *No* None, *OOH* Out-of-Hours

Data revealed a variable picture: those based in GP practice (GPs and primary care pharmacists) had relatively high levels of access. However, Clinical Nurse Specialists in particular reported limited access to others’ records, whether paper or electronic: 44% (173) reported no access to GP records and 66% were unable to access GP out-of-hours records.

Satisfaction with access to shared records to facilitate medicines access reflected these results: 39% (507) of respondents overall were either Not At All or only Slightly satisfied. Clinical Nurse Specialists and community pharmacists were especially likely to rate access as Not At All satisfactory, with half of all Clinical Nurse Specialists (50% 193) reporting that they were either Not At All or only Slightly satisfied.

#### Impact of nurse and pharmacist prescribing

Overall, just over half of respondents considered that prescribing by a nurse or pharmacist consistently had a beneficial impact on palliative care medicines access: Always 21% (283) or Often (34% 445). However, a quarter of GPs (25% 124), 29% (44) primary care pharmacists and 26% (58) of community pharmacists reported that they did not know about the impact of this initiative on access to medicines, with comments reflecting their lack of, or limited experience of these services.

#### Community pharmacy provision of palliative medicines

Table 5 shows the results of community pharmacist respondents’ opinions on issues identified in our review of the literature which may potentially impact their ability to facilitate access to medicines, confirming that a number of issues were problematic in practice.

**Table 5:** Issues influencing community pharmacy palliative medicines provision

How frequently do you:					
	Never	Rarely	Sometimes	Often	Always
Undertake medicines optimisation reviews for palliative care patients	61 (28)	65 (30)	52 (24)	26 (12)	13 (6.0)
Feel that lack of awareness of patients’ palliative status influences your ability to help patients access medicines	13 (6.0)	22 (10)	71 (33)	87 (40)	25 (11)
Encounter a discrepancy between palliative care medicines prescribed and the stock you hold	12 (5.5)	36 (17)	76 (35)	79 (36)	14 (6.5)
Limit your stock of palliative care medicines because ‘use by’ dates are likely to expire	27 (12)	37 (17)	47 (22)	67 (31)	40 (18)
Limit your stock of palliative care medicines because of lack of storage space	63 (29)	59 (27)	38 (18)	41 (19)	15 (6.9)
Experience problems receiving prescriptions electronically from patients’ GP practices	28 (13)	40 (18)	65 (30)	51 (23)	34 (16)
Encounter carers not having satisfactory ID, seeking to collect Controlled Drug prescriptions for patients	16 (7.3)	68 (31)	73 (33)	51 (23)	10 (4.6)

Responses range *n* = 215 to *n* = 217

Analysis of comments from 703 respondents to open-ended items on service delivery solutions highlighted the most frequently cited were: better community and primary care pharmacy provision (173/703); more nurse and pharmacist prescribers (162/703); shared electronic records (148 /703); and integrated working (145/703).

### Perceived effectiveness of service and impact of barriers

**Fig. 1:**
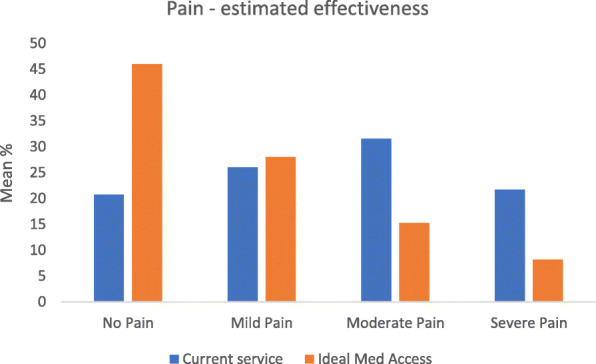
Mean proportions across pain category for current and improved medicines access

Respondents were asked to estimate the proportion of their patients who currently experienced No, Mild, Moderate and Severe pain (Q1), and then to re-estimate these proportions following removal of barriers to medicines access (Q2). Figure 1 shows that there would be a significant perceived improvement in pain control with better medicines access (No Pain *p* = .0018; Mild Pain p = NS; Moderate Pain *p* = .026; Severe Pain *p* = .047).

However, missing data for these questions was higher than for other items (18 and 23% respectively) and a further 10% for both provided invalid answers (that did not sum to 100).

There was a clear impact of respondent role in Q1 and Q2 data with pharmacists indicating they see higher proportions of patients with poor pain control. This was likely an artefact of ‘casemix’ or patients / their family carers who present to pharmacists, rather than service effectiveness. We tested the impact of various service factors on weighted Q1 response. After controlling for role there were few statistically significant predictors of pain control outcomes. However, respondents within services with access to GP records or Summary Care Records (an electronic record of patient information, created from GP medical records which can be seen and used by authorised staff in other areas of the health and care system involved in the patient’s direct care in England) [15] reported significantly lower pain estimates (*p* = .005 and *p* = .009 respectively).

## Discussion

### Main findings

This first large-scale survey of healthcare professionals providing palliative care to patients in the community in England captured views on medicines access and highlighted current practices and factors that operate to support or hinder access. Furthermore, although only indicative, our results provide evidence of the impact of access to medicines on patient outcomes (level of pain control in the example given).

GPs remain a predominant route for patients to access new prescriptions in working hours. However, nurses and, increasingly, primary care-based pharmacists are also actively contributing. This diversification of skill mix reflects a national policy drive to increase the range of healthcare professionals delivering primary care [16], including multi-practitioner palliative care [4].

However, our survey confirms there is potential to increase the numbers of nurses trained to prescribe palliative care medicines for patients, in particular Clinical Nurse Specialists. Less than half of Clinical Nurse Specialists surveyed were trained as prescribers, with costs and workplace support cited as inhibitory. Their potential to increase medicines access at end-of-life is clear from our results which show that, when trained as prescribers, Clinical Nurse Specialists were prescribing often, including controlled drugs, considered themselves highly competent, and were perceived by many as very effective at speeding patients’ access to medicines. Incentives are needed and barriers preventing training in this competency need to be removed in order to effectively support them, and perhaps also Community Nurses, to train to prescribe medicines. Such support should certainly include enabling all prescribing community-based nurses to utilise electronic prescribing systems, to speed safe prescription-writing and dispensing processes.

Internationally, the role of the pharmacist in the provision of health care in the community is growing rapidly, driven by GP workforce shortages and increasing population need [17]. Whilst many primary care pharmacists in our survey were trained to prescribe medicines and advising on and reviewing medicines with end-of-life patients, most did not feel competent prescribing in this area and prescribed infrequently. Whilst this self-selecting survey sample may over-represent engagement of primary care pharmacists in end-of-life provision, for those who are involved, being competent to manage palliative care medicines may provide patients with a further route to access the right medicines in a timely way, especially given primary care pharmacists’ level of access to shared records. In light also of the mixed levels of palliative care prescribing competence expressed by GPs in our survey, in line with others’ recommendations [18], this suggests a need for more training and / or support from palliative care specialists to underpin safe and confident prescribing for patients in the community. Education in end-of-life issues is crucial for the large number of doctors for whom end-of-life care is not the sole component of their work, but who play a significant role in caring for this group of patients [19].

National guidelines in England on end-of-life care recommend people and their carers should have access out-of-hours to a pharmacy service with medicines for symptom control [4]. Despite its positive evaluation by many in our survey, several factors are inhibiting the full potential of currently commissioned community pharmacy palliative care medicines services. Many lacked awareness of the service, and most services provided did not include access to medicines out-of-hours. Many pharmacists were not aware of patients’ palliative care status and stock issues also detracted from their ability to provide medicines to patients in a timely way. In line with others’ recommendations [20, 21], closer integration and better communication between community pharmacists and palliative care service providers is required to address such barriers, including for example, allowing community pharmacists access to palliative care registers of patients held locally by GPs [21].

A further, widespread barrier to the provision of swift and timely access to medicines in this context concerned a lack of shared access to patient records through which to communicate about medicines, especially for those outside of GP practice systems. Our survey respondents reported high levels of dissatisfaction with this aspect of service delivery. Access to patient records was also found to be the main determinant of services’ effectiveness on pain control. Prescribing will be delayed if the prescriber is not able to access patients’ clinical history and medications, thus potentially inhibiting symptom control and increasing patient and carer distress. To avoid out-of-hours medication safety incidents, Williams et al. (2018) [22] recommend communication solutions to enable primary care team members to collaborate effectively – such as encrypted end-to-end messaging systems embedded within the clinical record system – to allow appropriate plans for symptom relief to be coherently developed. Given the emphasis in recent national guidance [4] on multi-practitioner care, care co-ordination and health care professionals who can access patient records 24 h a day, 7 days a week, this lack of shared records access requires urgent attention.

### Strengths and limitations

To our knowledge, this is the first large scale survey internationally to provide data about healthcare professionals’ practices and views on the critical issue of how they currently provide access to medicines for palliative care patients in the community. As this was an on-line survey, we were not able to calculate response rates; however, our target numbers of respondents were exceeded in all but the Community Nurse group. We have no reason to suspect Clinical Nurse Specialist and community pharmacist samples were not nationally geographically spread. Survey respondents were assured of anonymity and we stressed our interest included understanding challenges and barriers, to encourage honest answers. Nevertheless, GP, primary care pharmacists, community pharmacist and Community Nurse respondents may have had a special interest in palliative care and therefore have atypical practices or views. The large sample size and low rate of missing data gives some reassurance that the conclusions of the study are not unduly affected by missing responses; however, there were notably higher rates of missing data for the questions on perceived effectiveness.

## Conclusions

Results show that a range of healthcare professionals and routes are currently being used to support patient access to palliative care medicines, but there is potential to increase the scale and effectiveness of these through addressing the barriers highlighted in this paper. These include increasing the competence of existing prescribers, training greater numbers of the community nurse workforce to prescribe using electronic systems, as well as ensuring better access to patient records across professional groups. This needs to take place alongside attention to out-of-hours service delivery, including improved awareness amongst GPs and pharmacists of existing service provision and tackling the impediments that currently reduce the effective provision of community pharmacy commissioned medicines services.

## Supplementary information

**Additional file 1.**

## Data Availability

The datasets used and/or analysed during the current study are available from the corresponding author on reasonable request.

## Abbreviation

GP: General Practitioner

## Footnotes

1 We define ‘access’ as patient and / or carer ability to obtain medicines from a prescriber and a dispenser together with any information and skills about medicines given at that point.

2 A number of standard medicines prescribed in advance - often in a specially marked container - specifically for the last days of life.
