# Supplementary material for: Supporting patient access to medicines in community palliative care: on-line survey of health professionals’ practice, perceived effectiveness and influencing factors
Source: BMC Palliat Care. 2020 Sep 24;19:148. doi: 10.1186/s12904-020-00649-3 (PMC7517636; doi:10.1186/s12904-020-00649-3)
Supplement: Supplementary file 1 — Additional file 1. [file 12904_2020_649_MOESM1_ESM.pdf]

ACTMED study

Page 1

ActMed Study:
Access to medicines during the last year of life

We would like to seek your views on providing patients and carers with access to medicines during the last year of life, and on what facilitates and prevents good practice. Your experience will contribute to a comprehensive overview of practice which will help shape national policy.

What do we mean when we refer to medicines access?

- **Medicines** - Regular and as necessary medicines, administered via all routes used for symptom management during the last year of life (**excluding** 'just-in-case' boxes).
- **Access** - prescribing, dispensing, supply and associated information-giving about medicines and information about access to medicines

The questionnaire will take approximately 10-15 minutes to complete. It is designed for all healthcare professionals, with supplementary questions for non-medical prescribers and community pharmacists.

If you have any queries about the research please contact Dr Natasha Campling, ActMed Senior Research Fellow, via email at actmed@soton.ac.uk

Thank you in advance for your help with this important study

Professor Sue Latter
Chief Investigator

This study has been funded by the NIHR Health Services and Delivery Research programme (HS&DR 16/52/23) and this survey is approved by the HRA (IRAS number 231837)

Please tick the box to consent to take part in this survey

- [ ] I agree to take part

Page 2

SECTION A: YOUR ROLE

A1 How would you describe your role?

- General practitioner (please go to Question A2)
- Clinical nurse specialist working from a hospice (please go to Question A2)
- Clinical nurse specialist employed by a Community Trust (please go to Question A2)
- Community nurse (RN) (please go to Question A2)
- Pharmacist
- Other role (please go to Question A2)

If other role, please describe

If you are a primary care pharmacist employed by GP practice(s), does this role include:

	Yes	No
Advising patients/carers about palliative care medicines	☐	☐
Medicines optimisation reviews for palliative care patients	☐	☐

Page 3

If you are a community pharmacist in a community pharmacy, do you provide an enhanced service for palliative care (e.g. on-demand availability of specialist drugs)?

- ○ Yes
- ○ No

If yes, does this service cover:

	Yes	No
Stocking a locally agreed list of core palliative care medicines	☐	☐
Out-of-hours availability of palliative care medicines from your pharmacy	☐	☐
Out-of-hours availability of palliative care medicines from other linked pharmacies in your area	☐	☐
Provision of information on the service to other pharmacy contractors and health care professionals in order that they can signpost patients to the service	☐	☐
Provision of information on the service to patients and carers directly	☐	☐

Is your pharmacy independent or part of a chain?

- ○ Independent
- ○ Small multiple
- ○ Large multiple

A2. Do you work out-of-hours?

- ☐ No
- ☐ Between 6.30pm - 8am weekdays
- ☐ Weekends/bank holidays

Page 4

SECTION B. ACCESS TO PALLIATIVE CARE MEDICINES PRESCRIPTIONS DURING WORKING HOURS

B1. Which of the following are you able to provide for patients to obtain *new prescriptions* during working hours? Please select all options *you* use:

	Yes	No	Not applicable
Personal home visits	☐	☐	☐
Telephone consultations	☐	☐	☐
E-mail consultations	☐	☐	☐
GP practice appointments	☐	☐	☐
Community pharmacy-based appointments	☐	☐	☐
Referral to a nurse prescriber	☐	☐	☐
Referral to a pharmacist prescriber	☐	☐	☐
Referral to a GP	☐	☐	☐

Other, please describe:

Page 5

B2. Overall, how satisfied are you with your ability to support patients in obtaining *new prescriptions* for palliative care medicines?

- Not at all
- Slightly
- Somewhat
- Very
- Extremely

B3. Are you aware of community pharmacies ('palliative care pharmacies') in your area that provide an enhanced service for palliative care (e.g. on-demand availability of specialist drugs)?

- Yes
- None providing services (please go to Section C)
- Not aware (please go to Section C)

B4. In your opinion, does an enhanced service for palliative care (on-demand availability of specialist drugs) via community pharmacies facilitate speed of access to medicines for patients?

- Yes
- No

Please give reasons for your response:

Page 6

B5. How often do you provide information on enhanced services for palliative care (e.g. on-demand availability of specialist drugs) to patients and carers?

- Always
- Often
- Sometimes
- Rarely
- Never

If you do not "always" provide information on enhanced services for palliative care to patients and carers, please give reasons for your response:

Page 7

SECTION C. ACCESS TO PALLIATIVE CARE MEDICINES OUT-OF-HOURS

C1. In your area, do palliative care clinical nurse specialists (CNSs) provide a 7 days a week community service between 9am - 5pm?

- ◯ Yes
- ◯ No
- ◯ Don't know

If yes, how effective is it at facilitating out-of-hours medicines access?

- ◯ Not at all
- ◯ Slightly
- ◯ Somewhat
- ◯ Very
- ◯ Extremely

Please give reasons for your response

Page 8

C2. When a patient requires medicine(s) arising from an out-of-hours consultation are they usually: *(please select one response that most accurately reflects usual practice)*

- ○ Given a PRESCRIPTION by the healthcare professional during the consultation
- ○ Referred to another healthcare professional for the PRESCRIPTION of the medicine
- ○ Other

If you selected Other, please specify:

C3. This question is about your ability to *signpost* services, rather than the quality of the services. Overall, how confident are you in your ability to advise patients/carers how best to access palliative care medicines out-of-hours?

- ○ Not at all
- ○ Slightly
- ○ Somewhat
- ○ Very
- ○ Extremely

Page 9

SECTION D. INFORMATION PROVISION ABOUT MEDICINES AT THE POINT OF ACCESS

D1. When a patient/carer is in need of a prescription, dispensing and/or supply of a palliative care medicine, how often do you give the following information?

	Always	Often	Sometimes	Rarely	Never
Possible side-effects of the medicine(s)	☐	☐	☐	☐	☐
How to manage possible side-effects of the medicine(s)	☐	☐	☐	☐	☐
How to tell if the medicine is working	☐	☐	☐	☐	☐
How to tell if the medicine is not working	☐	☐	☐	☐	☐
What to do if the medicine is not working	☐	☐	☐	☐	☐
Who to contact if changes to the medicines or new medicines are required	☐	☐	☐	☐	☐

Page 10

SECTION E. INTER-PROFESSIONAL COMMUNICATION ABOUT MEDICINES

E1. Do you have access to shared patient records for communication about medicines access between health professionals? Tick all that you are able to access:

	Yes - Paper	Yes - Electronic	No
GP records	☐	☐	☐
GP out of hours records	☐	☐	☐
Community nurse records	☐	☐	☐
Hospice/palliative care specialist records	☐	☐	☐
Community pharmacy records	☐	☐	☐
Summary Care Record	☐	☐	☐

Page 11

E2. In your service, is there a 'main' prescriber, who usually carries out the initial patient assessment, when a patient starts on a palliative care pathway?

- Yes
- No

If yes, what is their role?

- General practitioner
- Palliative care consultant
- Palliative care clinical nurse specialist
- Other

If you selected Other, please specify:

Is it communicated so that other professionals are clear who the main prescriber is?

- Yes
- No

E3. Overall, how satisfied are you with access to shared records to facilitate patient/carer access to medicines?

- Not at all
- Slightly
- Somewhat
- Very
- Extremely

Please give reasons for your response

SECTION F. NON-MEDICAL PRESCRIBING

F1. Are You a Non-Medical Independent Prescriber (nurse or pharmacist prescriber) (V300 qualification)?

- ○ Yes, actively prescribing as a non-medical prescriber
- ○ Yes, but not actively prescribing
- ○ Not a prescriber
- ○ Not a nurse or a pharmacist (e.g. general practitioner) -please go to question F6

If you are not actively prescribing please state the reasons for this:

If you are not a non-medical prescriber please state the reasons for this: (*Tick all that apply*)

- ☐ Cost of training
- ☐ Lack of employer/colleague support
- ☐ No backfill available
- ☐ No designated trainer
- ☐ Other

If you selected Other, please specify:

Page 13

If you are not a non-medical prescriber, please go to question F6

F2. Does your prescribing include controlled drugs?

- Yes
- No

F3. Are you able to prescribe via an electronic system?

- Yes, the system enables transfer of the prescription to the pharmacy
- Yes, but the system does not enable transfer of the prescription to the pharmacy
- No, prescribing pad only

F4. On average, how often do you prescribe palliative care medicines?

- Once a day or more
- 2-3 times a week
- Once a week or less
- Once a month or less

F5. Which palliative care medicine classes do you most frequently prescribe?

	Often	Sometimes	Rarely	Never
Analgesics	☐	☐	☐	☐
Anti-emetics	☐	☐	☐	☐
Laxatives	☐	☐	☐	☐
Antifungals	☐	☐	☐	☐
Corticosteroids	☐	☐	☐	☐
Hypnotics and muscle relaxants	☐	☐	☐	☐
Anti-muscarinics	☐	☐	☐	☐
Sedatives	☐	☐	☐	☐
"Specials" – unlicensed medicinal products	☐	☐	☐	☐

F6. Overall, how frequently does non-medical prescribing (prescribing by a nurse or pharmacist) have a beneficial impact on palliative care medicines access?

- ○ Always
- ○ Often
- ○ Sometimes
- ○ Rarely
- ○ Never
- ○ Don't know

Please give reasons for your response

Page 15

SECTION G. QUESTIONS FOR COMMUNITY PHARMACISTS

If you are not a community pharmacist please go to Section H

G1. Palliative care medicines access issues

How frequently do you:

	Always	Often	Sometimes	Rarely	Never
Undertake medicines optimisation reviews for palliative care patients?	☐	☐	☐	☐	☐
Feel that lack of awareness of patients’ palliative status influences your ability to help patients access medicines	☐	☐	☐	☐	☐
Encounter a discrepancy between palliative care medicines prescribed and the stock you hold	☐	☐	☐	☐	☐

Page 16

How frequently do you:

	Always	Often	Sometimes	Rarely	Never
Limit your stock of palliative care medicines because 'use by' dates are likely to expire	☐	☐	☐	☐	☐
Limit your stock of palliative care medicines because of lack of storage space	☐	☐	☐	☐	☐
Experience problems receiving prescriptions electronically from patients' GP practices	☐	☐	☐	☐	☐
Encounter carers not having satisfactory identification, seeking to collect controlled drug prescriptions for patients	☐	☐	☐	☐	☐

Page 17

SECTION H. OVERALL SERVICE PROVISION

H1. If you are a prescriber, how competent do you feel in prescribing palliative care medicines?

- Not at all
- Slightly
- Somewhat
- Very
- Extremely
- Not applicable

H2. Are there possible solutions to any gaps or problems with service delivery?

- Yes
- No

If yes, please describe

Page 18

We want to try and understand how effective you think the service you currently offer is. We are going to do this by asking you to think about pain management in particular.

H3. Out of a typical 100 palliative care patients during the last year of life who use your service, estimate the proportion with no pain, mild pain, moderate pain or severe pain at any one point in time.

Enter your estimates below and ensure the answers sum to 100:

No Pain

Mild Pain

Moderate Pain

Severe Pain

Page 19

H4. Imagine there were no barriers to medicines access in your service and all patients could receive the medicines they need quickly. What proportions of pain severity would there be with ideal medicines access?

Enter your estimates below and ensure the answers sum to 100:

No Pain

Mild Pain

Moderate Pain

Severe Pain

SECTION I. And FINALLY

I1. If you have any other comments to make about access to medicines at end-of-life please use this space:

I2. Please tell us the first part of your work postcode, so we can map the geographical spread of responses. This will be removed from the questionnaire so your data remain anonymous.

I3. If you work in a GP practice or community pharmacy please provide your organisation ODS code (sometimes known as a J code). This will be used to map your participation with your Clinical Research Network (CRN), and removed from the questionnaire so your data remain anonymous.

- ◯ Not applicable
- ◯ Don't know

ODS (or J) code:

Page 21

I4. In the next stage of the study we will be conducting case studies of medicines access experiences with patients, carers and healthcare professionals. Would you be willing to be contacted by the research team to discuss taking part in the next phase of our study?

○ Yes ○ No

I5. If yes, please give your name and email address. These will be removed from the questionnaire so your data remains anonymous.

Name:

Email address:

Thank you for taking the time to complete this survey

ActMed Study Questionnaire Version 1 (20.07.2018)

Page 22

Please return your completed survey to:

FREE POST RTXY-ZBLS-GJXL

Jakki Birtwistle, Research Fellow, Leeds Institute of Health Sciences,

Room 10.39, Level 10,

The Worsley Medical and Dental Building,

Clarendon Way, LEEDS LS2 9NL
